# Uroplakin IIIa Is a Marker in Bladder Cancer but Seems Not to Reflect Chemical Carcinogenesis

**DOI:** 10.1155/2018/8315410

**Published:** 2018-07-05

**Authors:** B. Szymańska, M. Matuszewski, J. Dembowski, R. Zdrojowy, A. Długosz

**Affiliations:** ^1^Department of Toxicology, Wroclaw Medical University, Wroclaw, Poland; ^2^Department of Urology and Oncological Urology, Wroclaw Medical University, Wroclaw, Poland

## Abstract

**Background:**

Uroplakins are glycoproteins investigated as potential markers of urothelial carcinoma. However, their role in chemical carcinogenesis is uncertain. In this study the diagnostic value of plasma and urine uroplakin IIIa (UPIIIa) levels in bladder cancer (BC) was investigated, particularly in the aspect of environmental exposure to chemical carcinogens, measured by DNA damage and detoxification ability in the BC smoking group. The correlation between uroplakin, 8-OHdG, and GST*π* was investigated.

**Material and Methods:**

This study included 61 BC patients and 33 healthy controls. UPIIIa, 8-OHdG, and GST*π* levels were estimated by the immunoenzymatic method (ELISA).

**Results:**

UPIIIa levels were elevated in BC patients in plasma (p≤0.001) and in urine (p≤0.001), as were 8-OHdG and GST*π* levels in urine. Moreover, the 8-OHdG level was higher in invasive or high grade tumors. A positive correlation between UPIIIa/GST*π* and 8-OHdG/GST*π* was observed, but no UPIIIa/8-OHdG correlation was noted.

**Conclusion:**

The study showed the diagnostic value of urine and plasma UPIIIa in BC (good sensitivity, specificity, and predictive value). The lack of UPIIIa correlation with 8-OHdG and smoking suggests that UPIIIa does not reflect the environmental exposure. The increased levels of 8-OHdG and GST*π* in the invasive tumor stage indicate their value in BC monitoring.

## 1. Background

The most common urinary bladder neoplasm is carcinoma of the urothelium. Urothelium is a highly specialized type of transitional epithelium. It consists of a few, mainly three to five, cell layers. Characteristic for this type of epithelium is high elasticity and low permeability. It is a barrier between urine and human tissue [1]. The main components of the external layer of urothelium are glycoproteins, uroplakins (UP). A few types of uroplakins are known (UPIa, UPIb, UPIIIa, and UPIIIb) [2]. They form advanced spatial structures described as asymmetric unit membrane (AUM), due to the fact of its appearance in transmission electron microscopy (TEM), which results from the presence of uroplakin in the outer leaflet of the membrane [3]. Approximately 90% of the urothelium surface is covered by AUM [4]. One of the roles of UP is improving urothelium tightness, which decreases the permeability to ions and substances dissolved in urine [5]. Some UP such as UPIa take part in initiation of infections by binding with the protein FimH of fimbria type 1 in* Escherichia coli*. This mechanism is crucial in bacterial adhesion to the host cellular membrane [6]. The destruction of urothelium causes release of proteins into blood and urine [7].

Carcinoma of the bladder urothelium represents approximately 90% of all bladder cancers. Worldwide, bladder cancer (BC) is the seventh most common cancer among men and the seventeenth in women. About 70-80% of malignancies are superficial and are treated by surgery. BC recurrence is observed in 50-70% of patients, and development to invasive disease can be observed in approximately 5-20% of patients [8]. In diagnosis of BC radiological methods (ultrasonography, computed tomography), cytological examination of urine and invasive procedures (cystoscopy, transurethral resection of bladder tumor (TURB-T)) are used. Some laboratory tests could also be helpful in BC diagnosis, e.g., BTA (bladder tumor antigen), which detects H-protein of the complement complex in urine of patients with BC. The ImmunoCyt test uses three types of monoclonal antibodies against mucin antigens and carcinoembryonic antigen (CEA) [9]. Other tests measure the level of nuclear matrix protein 22 (NMP22) or chromosomal aberrations (UroVysion test). The urological associations (American Urological Association, European Association of Urology) are skeptical about protein markers of bladder cancer, but the US Food and Drug Administration has approved some of them. Some reports have shown the possible usefulness of these markers [10, 11]. Even if cystoscopy cannot be replaced by another examination, there are some proteins, such as NMP22, which can substitute urine cytology [10]. Also the studies on new markers of BC among uroplakins are in progress [12–14].

BC is often classified as an environment-related neoplasm. The main risk factor of BC is tobacco smoking [12, 15]. Exposure to other carcinogens such as aromatic amines, polycyclic aromatic hydrocarbons, or arsenic could also lead to BC growth [12]. It has been established that carcinogenic xenobiotics conjugated with endogenic substances in the body are dissolved and excreted in urine. In the urinary bladder these complexes undergo partial hydrolysis which produces carcinogenic carbocations [16]. This process develops in people exposed to aromatic amines and could lead to BC [17]. Another study also showed higher BC morbidity in the population exposed to arsenic compounds in drinking water [18]. Another example of the influence of life style on BC development is smoking. Most BC patients are present or former tobacco smokers. Smoking is established as the main BC risk factor [12]. The final effect of exposure to carcinogenic xenobiotics depends on detoxification abilities in the body. Due to the listed facts it seems useful to search for new BC diagnostic markers, which could reflect the environmental impact and human detoxification parameters.

The aim of our study was to evaluate the diagnostic value of UPIIIa in plasma and urine of patients with BC, particularly in the aspect of environmental exposure to chemical carcinogens, measured by 8-OHdG level in the BC smoking group and the correlation with detoxification ability evaluated by the glutathione transferase *π* (GST*π*) isoenzyme level. The choice of noninvasive markers which reflect the environmental risk of carcinogens could improve the early diagnostics of BC. It is interesting to evaluate whether UPIIIa could be one of these markers.

Uroplakin IIIa is an isoform of UPIII isolated from AUM [19]. A recent study demonstrated a higher level of UPIIIa in urine of BC patients [11]. It was the first report which showed the possible usefulness of UPIIIa as a urine marker of BC. Other data have confirmed higher UPIII (not UPIIIa) concentration in serum of patients with BC [7]. According to our knowledge no research on UPIIIa in plasma has been reported. Furthermore, none of the published studies evaluated simultaneously UPIIIa in urine and blood, and none established the relation of UPIIIa to xenobiotic exposure and detoxification markers.

One marker which is reported to reflect the exposure to genotoxic xenobiotics is 8-hydroxy-2′-deoxyguanosine (8-OHdG) [20, 21]. The level of 8-OHdG in urine is reported as more specific for DNA damage than its level in blood. 8-OHdG is the most common product of oxidative DNA damage and is proportional to DNA damage. Many carcinogens disrupt oxidative balance by excessive production of reactive oxygen species (ROS), which plays an important role in pathogenesis of neoplasms. Some studies have shown that urine level of 8-OHdG could be an important marker of genetic damage due to xenobiotic exposure [22].

Glutathione transferase (GST) is an important detoxification enzyme. It plays a basic role in xenobiotics transformation, especially in conversion of aromatic hydrocarbons into mercapturic acid. GST*π* (an isoform of GST) is particularly involved in carcinogenesis. Our former studies have shown a statistically significantly higher urine level of glutathione S-transferase *π* (GST*π*) in BC patients [14, 23]. Moreover a twofold increase of GST*π* was observed in urine of BC patients in another study [24].

To achieve the aim of the study, we evaluated the levels of UPIIIa, 8-OHdG and GST*π* in BC patients in comparison to healthy controls (C) and also analyzed the differences in parameters between BC smokers and BC nonsmokers (environmental exposure to chemical carcinogens). The correlation between UPIIIa and listed markers was investigated by a mathematical method and also using a special test for diagnostic value. In order to perform the preliminary study of the prognostic value of listed markers, all results were related to BC stage (NMIBC and MIBC) and grade (low grade (LG) and high grade (HG)).

## 2. Materials and Methods

The study group is comprised of 61 BC patients of the Urology and Oncological Urology Department (Wroclaw Medical University), hospitalized between September 2014 and July 2015. The group consisted of 51 men (84%) and 10 women (16%), medium age 66 (41-88). All patients were informed about the study, participation was voluntary, and all signed written informed consent. The control group (C) collected by Biobank in Wroclaw is comprised of 33 healthy persons, 28 men (85%) and 5 women (15%). The percentage of smokers in the C group was the same as in the BC group. No statistically significant differences in characteristics between groups BC and C were found (Table 1).

Histopathological examination of tissue taken by TURB-T or radical cystectomy was performed in the Department of Pathomorphology and Oncological Cytology (Wroclaw Medical University). Based on histopathological results, patients were divided into subgroups, according to tumor stage T (TNM (Tumor Nodules Metastases), 2002) and grade (low grade/high grade, WHO/International Society of Urological Pathology, ISUP System 2004). The subgroups were Ta (n=28), T1 (n=18), T2 (n=4), T3 (n=5), TIS (n=6); low grade (LG; n=29) (53%); high grade (HG) in 32 (47%). Also subgroups of NMIBC (nonmuscle invasive bladder cancer: Ta+T1; n=46) and MIBC (muscle invasive bladder cancer: T2+T3+TIS; n=15) were selected (Table 1).

The material for laboratory tests was human blood and urine. In the BC group the material was obtained one day before surgical and pharmacological treatment. In the morning urine samples were collected in polystyrene containers (Aptaca, Italy) and then centrifuged for 10 minutes (1438xg at 4°C), and the obtained supernatant was removed to Eppendorf tubes and stored at −80°C for further investigation.

Blood samples were collected into plastic tubes (BD Vacutainer, Na_3_ citrate buffer, USA), with an anticoagulant. The tubes were centrifuged at 1438xg for at least 10 min at 4°C. The supernatant (plasma) was frozen at −80°C until being analyzed.

Our primary UPIIIa test showed its very low level in serum (in picograms). Due to this, tests were performed in plasma. The level of UPIIIa was measured in nanograms per plasma milliliter.

UPIIIa level was measured in urine and plasma by an immunoenzymatic (ELISA) Enzyme-Linked Immunosorbent Assay Kit, USCN Life Science Inc., PRC (designed by Cloud-Clone Corp. USA). GST-*π* was assessed in urine by Human Pi GST ELA-EKF Diagnostic and 8-OHdG was measured by Check Elisa JaJCA, Japan Institute for the Control of Aging.

The UPIIIa, 8-OHdG, and GST*π* levels in urine were calculated in relation to the urine creatinine level measured by Jaffe's routine method (under alkaline conditions creatinine reacts directly with picric ions (Picric Acid, SIGMA) to form a reddish complex, the absorbance of which can be measured at *λ*=520 nm.

UPIIIa (blood and urine) and 8-OHdG (urine) were determined in 61 patients, isozyme GST*π* (urine) in 41 of BC and in the control group C.

All patients completed a questionnaire about environmental risk factors of BC, e.g., smoking, place of residence, profession, and pesticide contact.

## 3. Measurement of Markers

### 3.1. UPIIIa

UPIIIa level Enzyme-Linked Immunosorbent Assay Kit, USCN Life Science Inc., was detected according to the manufacturer's instruction.

The microplate was precoated with an antibody specific to UPIIIa. Standards or samples (100 *μ*l) were added to the appropriate microplate wells with a biotin-conjugated antibody specific to UPIIIa. Next, avidin conjugated to horseradish peroxidase (HRP) was added to each microplate well and incubated (2 h, 37°C). Next TMB (3,3′,5,5′-tetramethylbenzidine) substrate solution was added, which caused that only those wells that contained UPIIIA, biotin-conjugated antibody, and enzyme-conjugated avidin displayed a change in color. The enzyme-substrate reaction was terminated by the addition of 0.1 M sulfuric acid solution and the color change was measured spectrophotometrically at a wavelength of 450 nm. The concentration of UPIIIA in the samples was then determined by comparing the absorption of the samples to the standard curve.

### 3.2. 8-OHdG and GST*π*

8-OHdG levels (Check Elisa JaJCA, Japan Institute for the Control of Aging) and GST*π* activity (Human Pi GST ELA-EKF Diagnostic, Ireland) were detected in urine using an enzyme-linked immunosorbent assay (ELISA) according to the manufacturer's instructions in the listed test.

## 4. Statistical Analysis

Statistical analysis was conducted with Statistica PL software (version 12.1). The normality of distribution was checked with the Kołomogorov-Smirnov test and the Lilliefors test. Student's t-test for parametric data and the Mann-Whitney U test for nonparametric data were used for variables. The values of p<0.05 were considered statistically significant. The associations between continuous variables were analyzed by Spearman's test for nonparametric data and Pearson's test for parametric data. Also, sensitivity, specificity, accuracy (ACC) of method, positive predictive value (PPV), negative predictive value (NPV), positive likelihood ratio (LR+) and negative likelihood ratio (LR-), and odds ratio (OR) were determined.

The receiver operating characteristic (ROC) curves were estimated. The area under the curve (AUC) and best cut-off point were calculated employing ROC analysis which evaluated the relation between sensitivity and specificity of markers.

The study was approved by the Ethics Committee of Wroclaw Medical University (KB-292/2-16). The participation was voluntary, respectful of human rights.

## 5. Results

### 5.1. Uroplakin IIIa

The mean urine level of UPIIIa was 2.44 ng/mg of creatinine (cr.) in the group of patients with BC and 1.02 ng/mg cr. in the control group. The difference was statistically significant (p≤0.001) (Table 2). The results of UPIIIa measurement without calculating the creatinine level were also higher than in the control group C. The mean plasma level of UPIIIa in BC patients (1.47 ng/ml) was higher than in the control group C (0.58 ng/ml) (p≤0.001). There was no statistically significant difference of UPIIIa level in either plasma or urine between nonmuscle invasive bladder cancer (NMIBC) and invasive bladder cancer (MIBC), but in both groups the UPIIIa level was higher than in the control C (p≤0.001). Similar results were obtained in LG and HG groups in BC. No significant difference in UPIIIa level in urine or plasma between low grade and high grade BC tumor was noted (Table 2). A significant difference between LG/control group and also HG/control was observed (Table 2). The comparison of HG/control and LG/control showed statistically significant differences (p≤0.001), which is interesting and indicates additional value of UPIIIa in LG diagnosis. No significant difference between UPIIIa level in BC smokers and nonsmoking patients was observed, in either urine or plasma (Table 2). Only between BC smokers and healthy controls was the difference significant in both urine and plasma (p<0.05). The examination of control group C showed a lack of difference between UPIIIa level in C smokers and C nonsmokers (Table 2). It confirms that smoking does not influence UPIIIa level in healthy people.

**Figure 1:**
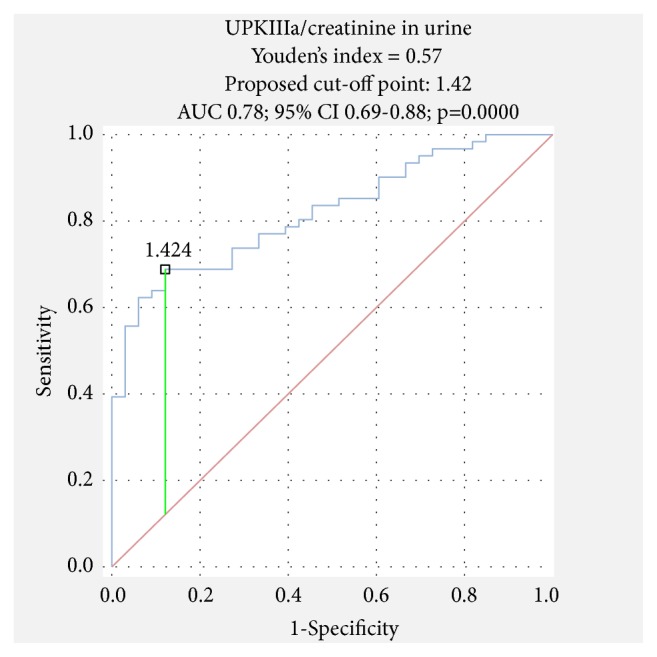
ROC analysis of UPIIIa in urine in BC group.

**Figure 2:**
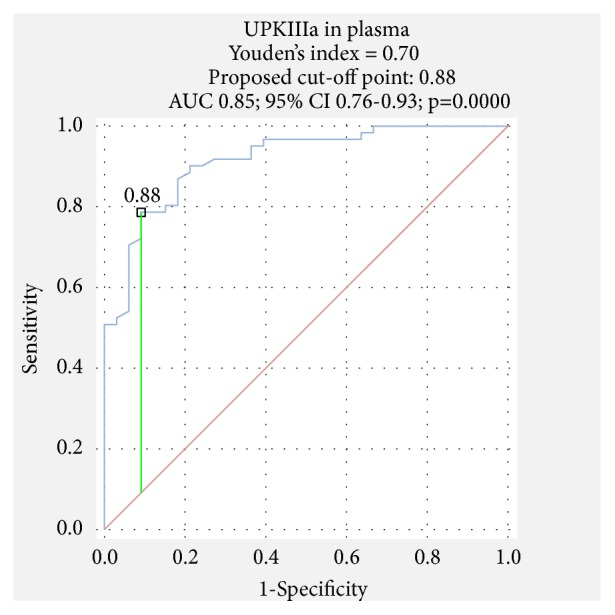
ROC analysis of UPIIIa in plasma in BC group.

The receiver operating characteristic (ROC) analysis was performed to estimate the diagnostic and prognostic value of UPIIIa. It was found that the measurement of UPIIIa level has a 69% sensitivity and 89% specificity in urine and a little higher in plasma (79%, 91%). The area under the curve (AUC) was calculated as 0.78 in urine (Figure 1) and 0.85 in plasma (Figure 2). It indicates good diagnostic value of UPIIIa in plasma (over 0.8).

The PPV was a little higher in plasma (0.94) but also high in urine (0.913) (Table 3). The results show that the level of UPIIIa measured by immunoenzymatic methods has good diagnostic value in bladder cancer. Also the prognostic value (PPV) seems to be high, but there are no significant differences in UPIIIa level according to grade and stage of BC.

### 5.2. 8-OHdG

The mean 8-OHdG level was 19.06 ng/mg cr. in the BC group, and it was significantly higher than in control group C (11.84 ng/mg cr.; p:0.003) (Table 4). Also a significant difference was observed between NMIBC (16.96 ng/ml cr.) and MIBC (25.48 ng/mg cr. p:0.013). Likewise 8-OHdG level was higher in the HG group (24.15 ng/mg cr.) than in the LG group (14.44 ng/mg cr.) p:0.001 (Table 4). Mean level of 8-OHdG in the group of smoking patients was 19.22 ng/mg cr. and 18.22 ng/mg cr. in nonsmokers. This difference between smoking and nonsmoking BC was not statistically significant.

Data shown in Table 4 indicate that the oxidative damage of DNA was more intensive in patients with advanced BC (MIBC).

### 5.3. GST *π*

The mean urine level of GST*π* in the BC group was 15.81 ng/mg cr. and was significantly higher than in control group C, 4.62 ng/mg cr. (p≤0.001). No significant differences in GST*π* level in urine were observed in comparison of NMIBC and MIBC between LG and HG groups. Mean level of GST*π* in the group of smoking patients (n=33, 80%) was 15.22 ng/mg cr. and it was 18.23 ng/mg cr. in nonsmokers (n=8, 20%) (Table 5).

### 5.4. Relationship between Markers

In order to evaluate whether UPIIIa reflects the exposure to chemical carcinogens, Spearman's rank correlation was calculated for UPIIIa, GST*π* and 8-OHdG, especially in BC smokers. Positive correlations were observed in urine between UPIIIa and GST*π* (correlation ratio R:0.34) and between 8-OHdG and GST*π* (R:0.35). No significant correlation was noted for UPIIIa and 8-OHdG either in urine or plasma in the whole BC group (Table 6) and in BC smokers (Table 7).

The absence of a relationship between UPIIIa and 8-OHdG indicated that UPIIIa is not a marker which reflects the environmental exposure. However, a correlation between UPIIIa and GST*π* showed its relevance in the detoxification process.

## 6. Discussion

Uroplakins are low molecular weight glycoproteins (15 to 47 kDa). Differences between uroplakins are based on the sequence of amino acids and number of transmembrane domains [4]. These proteins build the urothelial AUM [25]. UPIa and UPIb have 4 transmembrane domains but UPII and UPIIIa have only one domain whose C-end is turned into cytoplasm. The basic role of uroplakins is to form a barrier which protects tissue from substances dissolved in urine [4]. In mammals UPIII occurs in the isoforms UPIIIa, UPIIIb, and recently described UPIIIc. In contrast to UPIIIb (35 kDa), which is present in the urothelium, pericardium, and peritoneum, UPIIIa occurs only in the urothelium [4]. This knowledge was crucial to our choice of UPIIIa as a potential marker of BC.

Our preliminary research showed that UPIIIa concentration is higher in plasma than in serum. Due to this knowledge plasma was chosen as the material for research. Our study showed increased concentration of UPIIIa in plasma (p≤0.001) and in urine (p≤0.001) of BC patients in comparison to healthy control group C. Elevated UPIIIa level in urine of BC patients was previously described only by Lai et al. The results of this study showed high sensitivity (83%) and specificity (83%) of this marker (UPIIIa) in urine [11]. We noted lower sensitivity (69%) and higher specificity (88%) for UPIIIa in urine. The difference between our results and those reported before could be caused by different numbers of invasive and noninvasive causes in the examined groups. According to our knowledge there are no reports on UPIIIa level in plasma. The high sensitivity and specificity of UPIIIa in our study (79% and 91%, respectively) indicate its value in BC diagnosis.

Some reports have shown an increased amount of UPIII (without isoform consideration) in serum of BC patients. In this study the UPIII level was especially higher in MIBC (p≤0.001) than in NMIBC (p:0.040). Furthermore, this study emphasized that a higher serum UPIII level may be a prognostic marker with 66% specificity for MIBC and 33% for NMIBC [7]. The results of our study did not show a significant difference in isoform of uroplakin UPIIIa concentration in urine and plasma between NMIBC and MIBC patients. The obtained results also showed no significant difference between urine or plasma UPIIIa level according to tumor grade: LG or HG. Similar results were described previously in urine for G1, G2, and G3 classification [11]. Other research made on UPIII (not UPIIIa) showed a difference between tumor grade and UPIII concentration but in serum, not in urine (G1+G2 group versus G3 group; p:0.005) [7]. The lack of differences in urine or plasma UPIIIa level between invasive and noninvasive BC suggests that this isoform is not as good a marker in BC monitoring as serum UPIIIa level; however, further research is needed.

Bladder cancer is often classified as environmentally related. There is also abundant evidence that exposure to chemical carcinogens could cause BC. At first it was observed among azo-dyes workers exposed to aromatic amines and confirmed by others [26]. To date many other carcinogens have been found, such as arsenic or aromatic hydrocarbons [12]. The analysis of environmental risk factors for BC development indicates smoking as the most important cause of BC [12]. Also in our BC group the majority (77%) were former or actual smokers. Our comparison of UPIIIa level in BC smokers with BC nonsmokers did not show any difference either in urine or plasma between groups. This could lead to the conclusion that UPIIIa is not a marker which reflects the environmental risk. In order to obtain more information about the value of UPIIIa in chemical carcinogenesis the correlation of UPIIIa with 8-OHdG was examined. 8-OHdG is one of the markers of exposure to carcinogenic xenobiotics which generate ROS. It is a product of oxidative guanine transformation in DNA. Our previously described research on 8-OHdG level in urine of BC patients showed a positive correlation between smoking and 8-OHdG level [27]. There are many reports on increased 8-OHdG level in exposure to xenobiotics, e.g., in plasma of workers exposed to chloroaniline. The amount of 8-OHdG was higher in the smoking group than the nonsmoking one [28]. Other reports of glass production workers exposed to arsenic and some heavy metals showed increased 8-OHdG in urine [29, 30]. An interesting correlation between arsenic exposure on BC development and 8-OHdG level was observed in urine of rats treated with water containing dimethylarsenic acid (DMA) [31]. The research on BC showed an increased 8-OHdG level in cancer tissue [32, 33]. Prognostic value of 8-OHdG in BC was reported in another study, because a high level of 8-OHdG was related to poor prognosis [22]. Also our results indicate the prognostic value of 8-OHdG in BC. We observed higher urine expression of 8-OHdG in patients with HG than LG tumors (p:0.0129) and with MIBC than NMIBC (p:0.0011).

Glutathione S-transferases form a family of multifunctional proteins, which play an important role as detoxification enzymes in the second phase of biotransformation. Changes in expression of these proteins in some tissues, serum, or urine could be an important diagnostic detoxification index [34–36]. A higher level of GST*π* in cancer tissue in BC was previously reported [37]. Another study described a 2-fold higher GST*π* level in BC than in normal urothelium [24]. Our study shows a large increase in urine GST*π* level in BC in comparison to the control (p≤0.001). It confirms intensification of detoxification processes in BC patients and supports the value of GST*π* measurement in urine as a noninvasive marker in BC diagnosis.

One of our study aims was to evaluate UPIIIa as a potential marker of BC in the aspect of environmental exposure to carcinogenic substances (smoking). Spearman's rank correlation between smoking and markers was calculated to estimate the environmental influence. This problem has not been investigated before. The lack of correlation between UPIIIa and 8-OHdG in BC smokers suggests that UPIIIa does not reflect the exposure to carcinogens present in cigarette smoke.

## 7. Conclusion

The study showed the diagnostic value of urine and plasma UPIIIa in BC (good sensitivity, specificity, and predictive value). The lack of UPIIIa correlation with 8-OHdG and smoking suggests that UPIIIa does not reflect the environmental exposure. The increased level of 8-OHdG (also GST*π* level) in the invasive tumor stage indicates its value in BC monitoring.

## Figures and Tables

**Table 1:** Population characteristics.

**Population characteristic**	Patients (%)	Controls (%)
No.	61	33

Male	51 (84)	28 (85)

Female	10 (16)	5 (15)

Age, years	66 (41-88)	65(54-81)

Smokers	47 (77)	24 (73)

Nonsmokers	14 (23)	9 (27)

**Clinical staging**		

Ta	28 (46)	-

T1	18 (30)	-

T2	4 (6)	-

T3	5 (8)	-

TIS	6 (10)	-

**Clinical grading**		-

LG	29 (53)	-

HG	32 (47)	-

**Clinical subgroups**		-

NMIBC	46 (75)	-

MIBC	15 (25)	-

Ta, T1, T2, and TIS: subgroups of BC according to tumor stage T (TNM); LG: low grade; HG: high grade; NMIBC: nonmuscle invasive bladder cancer; MIBC: muscle invasive bladder cancer. TNM: Tumor Nodules Metastases, 2002.

**Table 2:** UPIIIa level in plasma and urine in BC and control group C.

Group	**UPIIIa in urine [ng/mg cr.]**			**UPIIIa in plasma [ng/ml]**			**p-value**	
	Mean	SD	Me	Mean	SD	Me	in urine	in plasma
C	1.02	0.54	0.98	0.58	0.31	0.65	**p≤0.001**	**p≤0.001**
BC	2.44	1.41	2.11	1.47	0.76	1.32	BC vs C	BC vs C

NMIBC	2.38	1.36	2.10	1.37	0.56	1.25	p=0.5978	p=0.4312
MIBC	2.64	1.59	2.23	1.77	1.15	1.32	NMIBC vs MIBC	NMIBC vs MIBC

LG	2.44	1.37	2.11	1.42	0.62	1.32	**p≤0.001 **	**p≤0.001 **
							LG vs C	LG vs C
HG							**p=0≤0.001 **	**p≤0.001**
	2.44	1.48	2.21	1.52	0.90	1.22	HG vs C	HG vs C
							p=0.9945	p=0.9939
							LG vs HG	LG vs HG

Non-smoking BC	2.02	1.11	2.02	0.96	0.61	0.96	p> 0.05	p> 0.05
Smoking BC	2.11	1.48	2.11	1.32	0.81	1.32	non-smoking BC	non-smoking BC
							vs smoking BC	vs smoking BC

Non-smoking C	1.28	0.41	1.17	0.68	0.33	0.69	p>0.05	p>0.05
Smoking C	0.94	0.56	0.91	0.553	0.30	0.65	non-smoking C	non-smoking C
							vs smoking C	vs smoking C

UPIIIa: uroplakin IIIa; C: control group; BC: patient group; NMIBC: nonmuscle invasive BC; MIBC: muscle invasive BC; LG: low grade; HG: high grade; SD: standard deviation; p: statistically significant difference; Me: median

**Table 3:** Diagnostic parameters of UPIIIa in bladder cancer.

	**UPIIIa/cr. in urine**	**UPIIIa/ in plasma**
Sensitivity	0.689	0.787
Specificity	0.879	0.909
PPV	0.913	0.941
NPV	0.604	0.698
ACC	0.755	0.83
LR+	5.68	8.656
LR-	0.354	0.234

PPV: positive predictive value; NPV: negative predictive value; ACC: accuracy; LR+: positive likelihood ratio; LR-: negative likelihood ratio.

**Table 4:** 8-OHdG levels in urine.

**Group**	**8-OHdG in urine [ng/mg cr.]**			
	Mean	SD	Me	p-value
BC	19.06	14.29	15.0	**p=0.0031** BC vs C
C	11.84	3.81	11.75	

NMIBC	16.96	11.96	13.68	**p=0.0129 ** NMIBC vs MIBC
MIBC	25.48	18.89	18.25	

LG	14.44	7.36	12.62	**p=0.0011** LG vs HG
HG	24.15	18.07	18.25	

Non-smoking patients	18.22	9.20	20.58	p>0.05 non-smoking vs smoking
Smoking patients	19.22	15.25	14.77	

UPIIIa: uroplakin IIIa; C: control group; BC: patient group; NMIBC: nonmuscle invasive BC; MIBC: muscle invasive BC; LG: low grade; HG: high grade; SD: standard deviation; p: statistically significant difference; Me: median

**Table 5:** GST*π* levels in urine.

**Group**	**GST** ***π*** ** in urine [ng/mg cr.]**			
	Mean	SD	Me	p-value
BC	15.81	21.08	8.68	**p=0.0005** BC vs C
C	4.62	3.43	3.48	

NMIBC	17.17	23.23	8.57	p=0.9606NMIBC vs MIBC
MIBC	10.19	5.34	9.12	

LG	12.80	16.99	7.91	p=0.1281LG vs HG
HG	20.05	25.75	13.71	

Non-smoking patients	18.23	37.58	7.79	p>0.05non-smoking vs smoking
Smoking patients	15.22	15.65	8.85	

UPIIIa: uroplakin IIIa; C: control group; BC: patients group; NMIBC: nonmuscle invasive BC; MIBC: muscle invasive BC; LG: low grade; HG: high grade; SD: standard deviation; p: statistically significant difference; Me: median

**Table 6:** Correlation ratio (R) between markers in BC group.

	UPIIIa [urine]	UPIIIa [plasma]	8-OHdG [urine]	GST*π* [urine]
UPIIIa [urine]	-	0.123	0.194	**0.344**

UPIIIa [plasma]	0.127	-	-0.042	-0.073

8-OHdG [urine]	0.194	-0.042	-	**0.349**

GST*π* [urine]	**0.344**	-0.073	**0.349**	-

**Table 7:** Correlation ratio (R) between markers in BC smokers.

	UPIIIa [urine]	UPIIIa [plasma]	8-OHdG [urine]	GST*π* [urine]
UPIIIa [urine]	-	0.175	-0.138	0.239

UPIIIa [plasma]	0.175	-	0.093	0.262

8-OHdG [urine]	-0.138	0.093	-	0.191

GST*π* [urine]	0.239	0.262	0.191	-

## Data Availability

The data used to support the findings of this study are available from the corresponding author upon request.
